# The critical clock – re-evaluating optimal timing of surgery for brainstem cavernous malformations

**DOI:** 10.1097/JS9.0000000000003291

**Published:** 2025-08-22

**Authors:** Yang Liu, Shuo Wang

**Affiliations:** Department of Neurosurgery, Beijing Tiantan Hospital, Capital Medical University, Beijing, China

*Dear Editor*,

Brainstem cavernous malformations (BSCMs) pose a formidable challenge to neurosurgeons due to their deep-seated location within the most eloquent part of the central nervous system^[1–3]^. Accounting for 19–30% of cerebral cavernous malformations (CCMs), these lesions are notorious for high hemorrhagic risk and devastating neurological consequences, even with minor bleeding events^[1,4,5]^. The natural history of BSCMs suggests a non-benign course: most patients present with symptomatic hemorrhage, often accompanied by cranial nerve deficits, hemiparesis, or life-threatening respiratory dysfunction^[1,4]^. Once a hemorrhage occurs, the risk of rebleeding is substantially elevated, particularly in the first 1–2 years^[4,5]^. Repeated hemorrhagic events not only worsen neurological deficits but also complicate surgical dissection, potentially leading to irreversible disability^[1,6,7]^.

Microsurgical resection remains the only definitive treatment to prevent further hemorrhage^[1,6]^. However, the optimal timing of surgery after a symptomatic hemorrhage has been debated for decades. Traditional neurosurgical wisdom, partly based on the Lawton–Garcia (LG) grading system, proposes acute (<3 weeks), subacute (3–8 weeks), and chronic (>8 weeks) intervention windows, suggesting that early surgery offers better long-term outcomes but may carry higher perioperative risks^[2,8]^. Until now, high-quality evidence quantifying this “critical clock” for BSCMs has been limited, leaving clinical practice largely dependent on institutional experience and expert consensus^[2,3,8]^.

In this issue, Li *et al* present a nationwide retrospective cohort study encompassing 293 patients who underwent microsurgical resection for hemorrhagic BSCMs in two high-volume neurosurgical centers in China[9]. Leveraging logistic regression modeling with restricted cubic splines and matching weight analyses, the authors investigated how hemorrhage-to-treatment time influences long-term neurological outcomes[9]. Their results challenge the notion that delayed surgery allows safer, more effective resection. Instead, they demonstrate that interventions performed beyond 42 days post-hemorrhage are associated with significantly worse functional outcomes, whereas acute (≤21 days) and subacute (22–42 days) interventions confer improved prognosis^[6,7,9]^. Notably, subacute intervention was associated with fewer cranial nerve deficits than acute surgery, suggesting a nuanced trade-off between early resection and surgical morbidity^[9,10]^.

Li *et al* identified three key observations from their analysis. (1) The timing of surgery is critical. Delaying intervention beyond 42 days after a hemorrhage was independently associated with a significantly increased risk of unfavorable long-term neurological outcomes (mRS > 2), whereas patients treated in the acute or subacute phase were more likely to achieve functional recovery^[6,7,9]^. (2) Subacute intervention may offer the optimal balance. While very early surgery (≤21 days) can prevent additional bleeding and potential clinical deterioration, procedures performed during the subacute window (22–42 days) were associated with fewer postoperative cranial nerve deficits and a lower risk of subtotal resection, likely due to improved hematoma liquefaction and clearer tissue planes facilitating safer dissection^[9,10]^. (3) Preoperative clinical burden predicts prognosis. Patients who experienced multiple hemorrhages or presented with severe baseline disability (mRS score of 5) were more prone to poor postoperative outcomes, underscoring the necessity of timely surgical decision-making before irreversible neurological damage occurs^[4,7,9]^.

The findings of this study have several noteworthy implications for neurosurgical decision-making in patients with hemorrhagic BSCMs. First, the identification of a critical treatment window of ≤42 days provides quantitative evidence supporting early-to-subacute intervention, moving beyond recommendations based solely on expert consensus^[3,6,9]^. These data have the potential to inform surgical triage, particularly in healthcare systems where referral delays are frequent and timely access to specialized centers is limited^[3,8]^.

Second, the study highlights the delicate balance between the risks and benefits of early intervention. Acute surgery offers the advantage of prompt decompression and prevention of recurrent hemorrhage; however, it is technically challenging due to friable tissue, poorly defined dissection planes, and a higher likelihood of subtotal resection^[2,10]^. In contrast, subacute intervention (22–42 days) appears to optimize operative safety by allowing partial hematoma resorption and better delineation of surgical planes, thereby potentially reducing morbidity while maintaining the benefits of early intervention^[9,10]^.

Third, postponing surgery beyond 42 days was associated with progressively worse neurological outcomes. This observation likely reflects ongoing secondary injury mechanisms – including gliotic scarring, axonal degeneration, and adhesions from repeated micro-hemorrhages – which can complicate resection and limit postoperative recovery^[4,6,7]^.

Finally, the study emphasizes the need for individualized decision-making rather than a rigid, one-size-fits-all timeline. Surgical planning should integrate hemorrhage-to-treatment time with patient-specific factors such as baseline neurological function, lesion size and location, presence of a developmental venous anomaly, and overall surgical risk profile to optimize outcomes on a case-by-case basis^[3,7]^.

This study provides valuable insights into the influence of hemorrhage-to-treatment time on surgical outcomes in patients with brainstem cavernous malformations (BSCMs); however, several limitations should be considered when interpreting these findings, each pointing toward specific directions for future research. First, the retrospective design inherently introduces selection bias, as surgical timing was primarily determined by clinical judgment and influenced by patient neurological status and stability^[6,9]^. Patients with severe deficits were more likely to undergo early intervention, whereas those with milder symptoms often experienced delays, potentially confounding the observed associations. To address this limitation, well-designed prospective multicenter studies, ideally randomized or pragmatic trials, are needed to validate the causal relationship between intervention timing and long-term neurological outcomes^[3,6,8]^. Second, inter-center variability in surgical expertise, operative techniques, perioperative care, and rehabilitation protocols may have contributed to heterogeneity in outcomes, complicating the attribution of differences solely to treatment timing^[3,6]^. Future research should therefore involve large-scale, collaborative studies with standardized surgical protocols and postoperative management to ensure reproducibility of results across diverse clinical settings and surgeon experience levels^[3,8]^. Third, despite the use of matching weights and multivariable adjustments, unmeasured confounders – such as hematoma evolution, intraoperative decision-making strategies, and surgeon-specific experience – could not be fully accounted for, leaving residual bias possible^[7,10]^. Incorporating advanced neuroimaging biomarkers, such as diffusion tensor imaging, tractography, and quantitative susceptibility mapping, as well as detailed intraoperative data, would help future studies better capture tissue injury, hematoma dynamics, and lesion complexity, thereby refining risk adjustment and improving analytic precision^[3,8]^. Fourth, the generalizability of these findings is limited because the data were derived from two specialized high-volume Chinese neurosurgical centers with rapid access to expert care^[3,9]^. Results may differ in low-volume institutions or resource-constrained healthcare systems^[3,8]^. International registries and multicenter collaborations encompassing diverse geographic and practice environments are essential to confirm whether the proposed ≤42-day threshold holds true globally and to adapt recommendations accordingly. Finally, as an observational study, causality cannot be firmly established, and the proposed timing threshold remains provisional. Longitudinal, prospective registries with standardized definitions for hemorrhage onset, intervention intervals, and outcome measures are necessary to enable reliable data pooling, facilitate robust meta-analyses, and ultimately inform consensus-driven, evidence-based clinical guidelines for optimal surgical timing in BSCMs^[3,8]^.

The study reviewed in this commentary enhances our understanding of how the timing of surgical intervention influences neurological outcomes in patients with hemorrhagic brainstem cavernous malformations. By identifying a critical treatment window within 42 days post-hemorrhage, it provides much-needed empirical evidence to inform surgical triage and optimize functional recovery^[6,9]^. These findings highlight the importance of timely decision-making in a pathology where repeated hemorrhages can lead to irreversible neurological damage^[1,4,6]^. However, translating these results into universally accepted clinical guidelines will require prospective validation, harmonized reporting standards, and collaborative international data sharing^[3,8]^. With further research, such evidence-based timing strategies have the potential to improve surgical planning, reduce long-term disability, and ultimately enhance quality of life for patients affected by this challenging vascular malformation^[3,8]^.

In preparing this correspondence, the authors adhered to the TITAN Guidelines 2025 on the responsible and transparent use of Artificial Intelligence in scientific writing and publication ethics^[11]^.

## Data Availability

Not applicable. This article does not generate or analyze any new datasets. Commentary on: Li Z, Lu J, Liu M, Ma L, Quan K, Zhang H, Liu P, Shi Y, Dong X, You C, Tian R, Zhu W. Association of hemorrhage-to-treatment time with outcomes in patients with brainstem cavernous malformations: a nationwide cohort study. International Journal of Surgery. April 2024. DOI: 10.1097/JS9.0000000000001111. PMID: 38668661

## Research registration unique identifying number (UIN)

Not applicable. This article does not report original research involving human participants.
